# Orthostatic Resiliency During Successive Hypoxic, Hypoxic Orthostatic Challenge: Successful vs. Unsuccessful Cardiovascular and Oxygenation Strategies

**DOI:** 10.3389/fphys.2021.712422

**Published:** 2021-10-27

**Authors:** Michael Nordine, Sascha Treskatsch, Helmut Habazettl, Hanns-Christian Gunga, Katharins Brauns, Petr Dosel, Jan Petricek, Oliver Opatz

**Affiliations:** ^1^Department of Anaesthesiology and Intensive Care Medicine, Berlin Institute of Health, Charité – Universitätsmedizin Berlin, Corporate Member of Freie Universität Berlin, Humboldt-Universität zu Berlin, Berlin, Germany; ^2^Center for Space Medicine and Extreme Environments Berlin, Berlin Institute of Health, Institute of Physiology, Charité – Universitätsmedizin Berlin, Corporate Member of Freie Universität Berlin, Humboldt-Universität zu Berlin, Berlin, Germany; ^3^Military University Hospital, Institute of Aviation Medicine, Prague, Czechia

**Keywords:** LBNP, hypoxia, cardiovascular, oxygenation, aerospace

## Abstract

**Introduction:** Rapid environmental changes, such as successive hypoxic-hypoxic orthostatic challenges (SHHOC) occur in the aerospace environment, and the ability to remain orthostatically resilient (OR) relies upon orchestration of physiological counter-responses. Counter-responses adjusting for hypoxia may conflict with orthostatic responses, and a misorchestration can lead to orthostatic intolerance (OI). The goal of this study was to pinpoint specific cardiovascular and oxygenation factors associated with OR during a simulated SHHOC.

**Methods:** Thirty one men underwent a simulated SHHOC consisting of baseline (P0), normobaric hypoxia (Fi02 = 12%, P1), and max 60 s of hypoxic lower body negative pressure (LBNP, P2). Alongside anthropometric variables, non-invasive cardiovascular, central and peripheral tissue oxygenation parameters, were recorded. OI was defined as hemodynamic collapse during SHHOC. Comparison of anthropometric, cardiovascular, and oxygenation parameters between OR and OI was performed via Student’s *t*-test. Within groups, a repeated measures ANOVA test with Holm-Sidak *post hoc* test was performed. Performance diagnostics were performed to assess factors associated with OR/OI (sensitivity, specificity, positive predictive value PPV, and odd’s ratio OR).

**Results:** Only 9/31 were OR, and 22/31 were OI. OR had significantly greater body mass index (BMI), weight, peripheral Sp02, longer R-R Interval (RRI) and lower heart rate (HR) at P0. During P1 OR exhibited significantly higher cardiac index (CI), stroke volume index (SVI), and lower systemic vascular resistance index (SVRI) than OI. Both groups exhibited a significant decrease in cerebral oxygenation (TOIc) with an increase in cerebral deoxygenated hemoglobin (dHbc), while the OI group showed a significant decrease in cerebral oxygenated hemoglobin (02Hbc) and peripheral oxygenation (TOIp) with an increase in peripheral deoxygenated hemoglobin (dHbp). During P2, OR maintained significantly greater CI, systolic, mean, and diastolic pressure (SAP, MAP, DAP), with a shortened RRI compared to the OI group, while central and peripheral oxygenation were not different. Body weight and BMI both showed high sensitivity (0.95), low specificity (0.33), a PPV of 0.78, with an OR of 0.92, and 0.61. P0 RRI showed a sensitivity of 0.95, specificity of 0.22, PPV 0.75, and OR of 0.99. Delta SVI had the highest performance diagnostics during P1 (sensitivity 0.91, specificity 0.44, PPV 0.79, and OR 0.8). Delta SAP had the highest overall performance diagnostics for P2 (sensitivity 0.95, specificity 0.67, PPV 0.87, and OR 0.9).

**Discussion:** Maintaining OR during SHHOC is reliant upon greater BMI, body weight, longer RRI, and lower HR at baseline, while increasing CI and SVI, minimizing peripheral 02 utilization and decreasing SVRI during hypoxia. During hypoxic LBNP, the ability to remain OR is dependent upon maintaining SAP, via CI increases rather than SVRI. Cerebral oxygenation parameters, beyond 02Hbc during P1 did not differ between groups, suggesting that the during acute hypoxia, an increase in cerebral 02 consumption, coupled with increased peripheral 02 utilization does seem to play a role in OI risk during SHHOC. However, cardiovascular factors such as SVI are of more value in assessing OR/OI risk. The results can be used to implement effective aerospace crew physiological monitoring strategies.

## Introduction

Rapid environmental changes can occur during aerospace travel, which can have significant consequences for crew health and safety. Specific environmental changes include acute alterations in atmospheric composition, such as hypoxia during sudden loss of cabin pressure for example, as well as orthostatic challenges, such as during take-off, orbital re-entry, and aerobraking maneuvers. Taken as solitary events, innate physiological counter-responses can compensate for these challenges, allowing the crew enough time to enact exogenous countermeasures, such as utilizing supplemental oxygen, or adjusting the approach vector to lessen the degree of hyper-gravity. Combined hypoxia and orthostatic challenge would present as a significant challenge for the innate physiological mechanisms to overcome, as these challenges require unique responses. Remaining orthostatically resilient (OR) during such intense environmental challenges, is essential, and any crew member that exhibited orthostatic intolerance (OI) during an acute onset hypoxic orthostatic challenge would be unable to perform complex motor or cognitive tasks, thereby increasing the risk of critical mission failure.

OR during a successive hypoxic-hypoxic orthostatic challenge (SHHOC) is dependent upon precise orchestration of physiological counter-responses to ensure cerebral perfusion and oxygenation. Under normal atmospheric conditions, cerebral blood flow and oxygenation are maintained via cerebral autoregulation. Cerebral autoregulation is, however, sensitive to changes in 02/C02. During acute hypoxia, vasodilation of the cerebral vessels is known to occur (Hoiland et al., 2016). This cerebral vasodilation increases cerebral blood flow, and thus provides the brain with an ample supply of oxygenated blood to maintain an optimal 02 extraction/utilization (Van Mil et al., 2002). States of hypoxia can alter cerebral autoregulation, so that optimal cerebral perfusion is impaired, despite compensatory cardiovascular reactions (Iwasaki et al., 2011). In the macro-circulation, hypoxia induces a compensatory increase in cardiac output (CO) via increases in stroke volume (SV) and heart rate (HR), with reductions in systemic vascular resistance (SVR) (Paparde et al., 2015; Siebenmann and Lundby, 2015). This response is modulated by the peripheral chemoreceptor system, located in the carotid bodies and aortic arch (Kane et al., 2020) and ensures adequate cerebral 02 delivery (D02) (Siebenmann and Lundby, 2015). Furthermore, a compensatory capillary recruitment occurs during acute hypoxia, leading to a significant decrease in SVR, thus allowing for increased tissue perfusion and 02 extraction (Mirna et al., 2020). A non-optimal response to hypoxia would include a decrease in CO, and an upsurge in SVR, which would lead to an increased peripheral 02 consumption, and inadequate blood flow. This non-optimal response could lead to compromised cerebral perfusion and oxygenation, thus increasing the risk for an OI event during an orthostatic challenge.

During acute orthostatic challenges, a sudden decrease in central blood volume, via cranial to caudal shifts, occurs. This in turn activates the baroreceptor reflex (Melchior et al., 1992), which activates a compensatory increase in SVR and HR, to maintain mean arterial pressure (MAP) and cerebral perfusion. Maintaining MAP above 65 mmHg is presumed to be the critical threshold that can preserve cerebral autoregulation, however, once MAP is below < 65 mmHg, cerebral autoregulation fails, and cerebral blood flow is then compromised (Rickards, 2015). To remain OR during an orthostatic challenge, a balanced HR and SVR response are needed, which preserve CO and critical MAP. If these HR and SVR increases are not optimal nor carefully orchestrated, a critical decrease in cerebral perfusion can occur, thus leading to OI (Manuel et al., 2020).

The number of studies examining successful/unsuccessful response patterns during SHHOC are few. The only study to our knowledge, found that hypoxia blunts an effective SVR response during hypoxic orthostatic challenge, and the maintenance of cerebral perfusion is thus reliant upon maintaining CO to maintain MAP (Shepherd et al., 2020). Other studies have found that acute hypoxia can augment baroreflex responses as well as increase sympathetic outflow, meaning that acute hypoxic exposure, prior to and during orthostatic stress, would increase the chance of OR (Halliwill et al., 2003). Aarts et al. (2017) found a 70% OI occurrence during hypoxic LBNP, and that OR during hypoxic LBNP is reliant upon maintaining a MAP above 70 mmHg. Furthermore, that study found that the OI group was unable to mount an effective HR response during hypoxic LBNP (Aarts et al., 2017). Other working groups have concluded that the ability to maintain OR during SHHOC is dependent upon appropriately timed and orchestrated neural recruitment strategies (Badrov et al., 2015), and a misguided activation of the endogenous physiological counter-measure reactions could lead to OI. The ability to remain OR during a hypoxic orthostatic challenge would be dependent upon a robust cardiac (HR and CO) response, as hypoxia can attenuate any effective SVR counter-response. Finally, no study has examined both cardiovascular and central/peripheral oxygenation reactions during a SHHOC, so any successful/unsuccessful cerebral and peripheral oxygenation strategies are unknown at this time.

This study was performed to comprehensive analyze and catalog successful vs. unsuccessful cardiovascular and oxygenation strategies involved in maintaining OR during SHHOC. Given the findings of previous studies and the degree of orthostatic stress, we expected an OI rate of at least 50%, and that crew members exhibiting increases in CO (via HR increases and preserved SV) and decreases in SVR during hypoxia would exhibit a greater degree of OR during subsequent hypoxic orthostatic challenge. Also, the ability to remain OR during hypoxic orthostatic stress should primarily rely on maintaining CO as opposed to increases in SVR, to preserve cerebral oxygenation and perfusion. We further hypothesized that cerebral oxygenation and peripheral oxygenation parameters such as 02 utilization/extraction, could offer unique insights into OR/OI status. Finally, our goal was to determine which baseline factors, as well as physiological factors could be used as a predictive factor in pinpointing OR/OI status. The results would be therefore highly beneficial for, implementing appropriate physiological monitoring strategies for aerospace crews.

## Materials and Methods

### Environmental Challenge Simulation

This study took place in September–October 2013, at the Prague military hospital, division of aerospace medicine, Prague, Czech Republic. A total of 31 Czech military aviation students underwent this SHHOC trial as part of their advanced military aviation training curriculum. Since this course was a mandatory part of the curriculum, no recruitment of volunteers was needed. All participants gave their informed consent for additional monitoring as part of this study, and the division of aerospace medicine approved of the study. The SHHOC consisted of a three-stage environmental challenge course. Phase 0 (P0), a 5-min sitting upright baseline phase, was followed by a normobaric hypoxic phase (P1). Normobaric hypoxia (Fi02 12%) was induced via facemask and a hypoxic gas mixture containing 12% Fi02. P1 lasted until central oxygen saturation (Sp02c) fell to 85%. Phase 2 (P2) consisted of a rapid onset of −70 mmHg lower body negative pressure (LBNP) for either 60 s or until OI occurred. OI was defined as either a narrowing of pulse pressure (PP), a 20% decrease in baseline mean arterial of systolic arterial pressure (MAP/SAP), a significant decrease in heart rate (HR < 60 bpm), loss of consciousness (LOC) or responsiveness (LOR), as well as reports of blurred vision, slurred speech, or vegetative symptoms such as sweating. The LBNP was turned off, mask removed, and normal atmospheric conditions were re-established if any participant exhibited any OI criteria. OR was classified as 60 s of hypoxic LBNP exposure without incident. The flight physician sat next to the participant the entire time and could stop the LBNP at any point.

On the day of testing, which was assigned randomly to each participant, a “check-in” was performed, which included acquiring the height and weight of each subject. Also, each participant had to dress down to boxer shorts, and the LBNP-seal was fitted across the waist, superior to the iliac crest. Then, each participant sat at a 90-degree upright position in the LBNP device while all monitoring equipment was attached.

### Anthropometric Measurements

A height in centimeters (cm) and weight in kilograms (kg) was recorded for each participant. From this, a body mass index (BMI kg/cm^2^), body surface according to Dubois (BSA cm^2^), along with an estimated lean body mass according to Boer (LBM kg) were all calculated.

### Hemodynamic Monitoring

Hemodynamic monitoring was performed non-invasively using the Finapress device using Beatscope software (^®^Finapres Medical Systems, Netherlands). SAP, DAP, and MAP were continuously measured via the dominant hand via a pneumatic pump placed on the index and middle finger. HR was measured via 3 Lead ECG, with R-R Interval (RRI) being taken from Lead II from each participant. Stroke volume (SV) was measured using pulse contour analysis, through which a cardiac output (CO) was calculated. Systemic vascular resistance (SVR) was calculated via MAP/CO. CO, SV, and SVR were all divided by BSA to give cardiac index (CI), stroke volume index (SVI), and systemic vascular resistance index (SVRI).

### Central and Peripheral Oxygenation Parameters

Central 02 oxygenation (Sp02c) was measured via ear clip, while a finger probe was used to measure Sp02 peripherally from the non/dominant finger (Sp02p). Central and peripheral oxygenation was measured via near infrared spectroscopy (NIRS) (NIRO 200, Hamamatsu Instruments, Hamamatsu, Japan). Cerebral oxygenation was measured with a NIRS probe fixated to the right forehead, while peripheral oxygenation was measured with a second NIRS probe affixed to the right lateral thigh, bisecting the vastus lateralis muscle. The complete NIRS measurement included central and peripheral tissue oxygen index (TOIc/TOIp), delta central and peripheral total (tHbc/tHbp), oxygenated (02Hbc/02Hbp), and deoxygenated hemoglobin (dHbc/dHbp).

### Data Analysis

Upon conclusion of the study, cardiovascular and oxygenation parameters were synchronized, compressed into a data table, and grouped according to the corresponding phases. Beyond baseline, all values except Sp02c/p were converted to delta from P0 to highlight the magnitude of physiological adaptation during P1 and P2, and to reduce the effect of inter-individual variability. Groups were classified as OR and OI based on LBNP time (< 60 s = OI, 60 s = OR). P0 values were averaged over a 5-min time span. P1 values were averaged from the point of reaching Sp02c of circa 85%. P2 values were taken from the last 10 s of LBNP exposure, to highlight maximal effect. Group comparison was performed via Student’s *t*-test on a phase per phase basis. Within group analysis was performed via repeated measures ANOVA with Holm-Sidak *post hoc* test. Cardiovascular and NIRS data was synchronized using R software (© The R Foundation). Statistics were performed via JASP statistical software (Version 0.13.1), and graphics were created using Data graph software for Mac OS (Version 4.6.1). All parameters are reported as mean ± SEM. This study was exploratory in nature, so that a specific power test was not performed prior to statistical analysis. A significance level of *p* < 0.05 was used for all statistical testing. A sensitivity, specificity, positive predictive value (PPV), and odd’s ratio analysis (OR) were performed for parameters that were significantly different between OR/OI, and from P0, to assess the predictive capability of these parameters to predict OR/OI.

### Results: Overall and Baseline

From the 31 participants, 9 (29%) could be classified as OR, whereas 22 (71%), were classified as OI. One participant exhibited LOC during P1, and had his exposure terminated by the flight physician prior to P2. This data from this participant was used for baseline and P1 analysis. Average LBNP exposure time for the OI group was 25.1 ± 3.5 vs. 60 s for the OR group. At baseline, the OR group had significantly greater BMI, weight, RRI, and Sp02p, and significantly lower HR. No other significant differences at baseline were revealed between both groups. Baseline values are displayed in Table 1.

**TABLE 1:** Baseline (P0) anthropometric, cardiovascular, and central/peripheral oxygenation parameters between OR and OI.

**Parameter**	**OR (*n* = 9)**	**OI (*n* = 22)**
Age (years)	252.0	241.2
Height (cm)	1833.1	1821.6
Weight (kg)	824.6*	731.9
BMI (kg/m^2^)	241.0*	220.4
BSA (m^2^)	2.00.7	1.90.03
Est. LBM (kg)	632.7	591.2
MAP (mmHg)	1043.6	1052.3
SAP (mmHg)	1374.0	1383.4
DAP (mmHg)	832.9	841.7
CI (L/min/m^2^)	4.00.3	4.50.2
SVI (ml/m^2^)	472.3	461.6
HR (beats/min)	865.4	1003.1*
RRI (ms)	72248.1*	62218.8
SVRI (mmHg/min/L/m^2^)	27.02.2	24.01.3
Sp02c (%)	980.4	980.2
Sp02p (%)	980.3*	970.3
TOIc (%)	691.6	701.1
TOIp (%)	661.8	691.2

*p < 0.05.

### Orthostatically Resilient vs. Orthostatic Intolerance: P1 and P2 Differences

**FIGURE 1:**
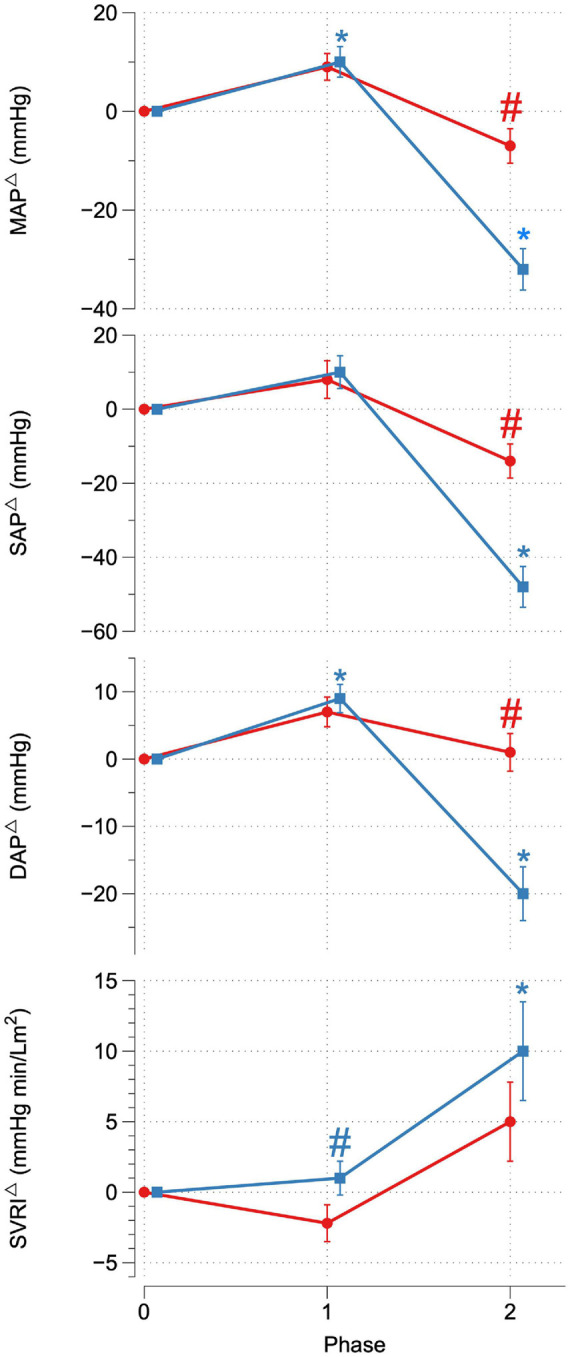
Delta MAP, SAP, DAP, and SVRI for OR (red) and OI (blue) during SHHOC. ^#^Significant difference between groups. *Significant change from P0.

**FIGURE 2:**
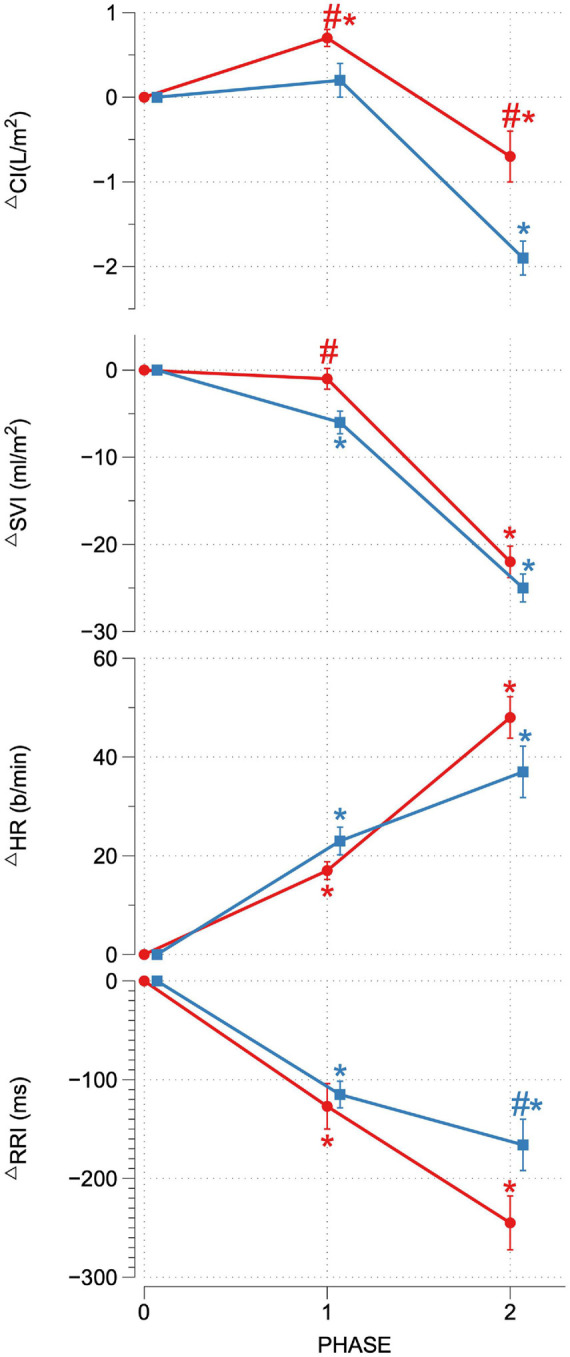
Delta CI, SVI, HR, and RRI for OR (red) and OI (blue) during SHHOC. ^#^Significant difference between groups. *Significant change from P0.

Upon reaching peak hypoxia (circa 85% Sp02c), the OR group exhibited a significantly greater increases in CI, SVI, and decrease in SVRI compared with the OI group. No other significant differences between the groups were observed during P1. During P2, the OR group exhibited a significantly greater delta SAP, DAP, MAP, and CI as well as a stronger decrease in RRI compared to the OI group. No differences in central or peripheral oxygenation were found between both groups throughout the SHHOC. Figures 1, 2 highlight cardiovascular trends throughout the study for OR/OI groups, while information regarding central and peripheral oxygenation is found in Table 2.

**TABLE 2:** Central and peripheral oxygenation trends for OR/OI during P1 and P2.

**Parameter**	**OR P1**	**OI P1**	**OR P2**	**OI P2**
ΔTOIc (%)	−5.30.5*	−6.40.5*	−10.82.6*	−9.40.8*
ΔtHbc (μmol/L)	2.41.1	2.70.6	−0.91.0	−1.51.1
Δ02Hbc (μmol/L)	−2.31.1	−3.00.4*	−6.91.1*	−7.60.8*
ΔdHbc (μmol/L)	4.70.3*	5.60.4*	6.10.6*	6.10.7*
Sp02c (%)	830.4*	830.4*	810.7*	810.8*
ΔTOIp (%)	−1.40.5	−2.30.5*	−5.11.1*	−4.80.7*
ΔtHbp (μmol/L)	0.50.6	0.90.5	13.41.4*	13.51.4*
Δ02Hbp (μmol/L)	−0.30.9	−1.20.6	5.21.8*	4.40.8*
ΔdHbp (μmol/L)	0.80.5	2.10.5*	8.20.5*	9.11.1*
Sp02p (%)	810.9*	800.9*	821.2*	801.1*

*Significant change from baseline.

### Orthostatically Resilient and Orthostatic Intolerance: Within Group Reactions, P1 and P2

Taken as a group, cardiovascular reactions amongst the OR group during P1 included significant HR upsurges, leading to a significant CI increase, with stable SVI, as well as a significant decrease in RRI. TOIc significantly decreased, while dHbc increased. All other central and peripheral oxygenation parameters did not significantly deviate from baseline levels during P1. Cardiovascular changes during P2 for the OR group included a further significant HR increase, while CI, SVI, and RRI decreased from P0. MAP, SAP, DAP, and SVRI did not significantly change from P0. During P2, TOIc, 02Hbc, and TOIp all decreased significantly from P0, while dHbc, 02Hbp, dHbp, and tHbp all increased significantly from P0. Sp02c/p were significantly lower in P1 and P2 compared to P0.

For OI, the following cardiovascular changes occurred during SHHOC: a significant increase in MAP, DAP, and HR, with a concomitant significant decrease in SVI and RRI during P1. Amongst oxygenation parameters, TOIc, 02HBc, and TOIp all significantly decreased, while dHbc, and dHbp all increased significantly from baseline. During P2, MAP, SAP, DAP, CI, SVI, and RRI all significantly decreased from baseline, while HR and SVRI significantly increased from P0. TOIc, TOIp, and 02Hbc continued to decrease from P0 levels. 02Hbc did not change significantly from baseline, while dHbc remained elevated from P0 levels. Sp02c/p were significantly lower during P1 and P2 compared with P0.

### Orthostatically Resilient/Orthostatic Intolerance Performance Diagnostics

Performance diagnostics performed for significant factors between groups and within groups per phase are displayed in Tables 3–5. From the baseline anthropometric factors, body weight and BMI showed identical sensitivity, specificity, and PPV, with weight having a greater OR (0.92) than BMI (0.61). Baseline hemodynamic and oxygenation factors of importance were, RRI, HR, and Sp02p, with RRI showing the highest sensitivity (0.95), Sp02p the highest specificity (0.33), comparable PPV (0.74, 0.74, and 0.76), while HR and RRI having the highest OR (1.07 vs. 0.99). During P1, delta SVI, CI, SVRI, and dHbp showed high sensitivity (0.95), while specificity was higher in CI and SVI (0.44). Both delta CI and SVI exhibited a PPV of 0.80, Delta SVRI and dHbp had a sensitivity of 0.91, however, low specificity and PPV, with albeit higher OR (1.33 and 1.36). Performance diagnostics for P2 revealed that delta SAP had the highest sensitivity, specificity, PPV, and odd’s ratio compared to delta MAP, DAP, CI, and RRI.

**TABLE 3:** Performance diagnostics and odd’s ratio for P0 significant factors.

**Parameter**	**Sensitivity**	**Specificity**	**Positive predictive value**	**Odds ratio**	***P*-value**	**Confidence interval**
Weight (kg)	0.95	0.33	0.78	0.92	0.06	0.84–1.00
BMI (kg/m^2^)	0.95	0.33	0.78	0.61	0.04	0.4–0.97
HR (b/min)	0.91	0.22	0.74	1.07	0.05	1.0–1.14
RRI (ms)	0.95	0.22	0.75	0.99	0.05	0.98–1.0
Sp02p (%)	0.90	0.33	0.76	0.37	0.04	0.14–0.95

**TABLE 4:** Performance diagnostics and odd’s ratio for P1 significant factors.

**Parameter**	**Sensitivity**	**Specificity**	**Positive predictive value**	**Odds Ratio**	***p*-value**	**Confidence interval**
ΔCI (L/min)	0.91	0.44	0.80	0.09	0.03	0.0–0.79
ΔSVI (ml/m^2^)	0.91	0.44	0.80	0.8	0.03	0.65–0.98
ΔSVRI (mmHg/L/m^2^)	0.91	0.22	0.74	1.33	0.05	1.00–1.77
ΔdHbp (μmol/L)	0.91	0.00	0.71	1.36	0.17	0.88–2.11

**TABLE 5:** Performance diagnostics and odd’s ratio for P2 significant factors.

**Parameter**	**Sensitivity**	**Specificity**	**Positive predictive value**	**Odds ratio**	***P*-value**	**Confidence interval**
ΔMAP (mmHg)	0.86	0.67	0.86	0.86	0.02	0.76–0.97
ΔSAP (mmHg)	0.95	0.67	0.87	0.90	0.02	0.83–0.98
ΔDAP (mmHg)	0.86	0.44	0.78	0.88	0.02	0.8–0.98
ΔCI (L/min/m^2^)	0.90	0.67	0.86	0.14	0.02	0.03–0.70
ΔRRI (ms)	0.86	0.22	0.72	1.01	0.07	1.0–1.02

## Discussion

### Overall Results

Upon conclusion of this SHHOC study, it was found that 71% (*n* = 22) of the crew experienced OI during SHHOC, whereas 29% (*n* = 9) remained OR. The OR group had a greater body weight, BMI, Sp02p, lower HR, and longer RRI than the OI group at P0. During P1, the OR group exhibited a significantly greater increase in CI, SVI, and lower SVRI, compared to the OI group. During P2, the OR group maintained a significantly greater MAP, SAP, DAP, CI, with a shorter RRI than their OI counterparts. No central or peripheral oxygenation differences were found between groups. The only distinguishing oxygenation factors, was that of significant decrease in 02Hbc and TOIp, as well as an increase in dHbp in the OI group during P1. This finding suggests a decreased cerebral 02 supply combined with an increased cerebral 02 utilization, and an increased peripheral oxygenation usage in this group during P1, which may have predisposed this group for a OI during P2. Total body weight and BMI at baseline had the highest sensitivity, PPV, and odd’s ratio of the significant baseline factors, although a low specificity was observed. Furthermore, delta SVI during P1 was the factor that had the highest sensitivity, specificity, and PPV compared with delta CI and SVRI. Finally, delta SAP was the main cardiovascular factor most associated with OR during hypoxic LBNP.

The results of this study can confirm that the ability to maintain OR during combined hypoxic orthostatic stress presents as a formidable challenge to the innate physiological counter-response system, and our results are in line with other studies showing that OI occurrence during such an event range from 30 to 70% (Rowell and Seals, 1990; Crandall et al., 2019). The physiological basis of this is thought to be due to excess concentrations of serum adrenaline, as opposed to noradrenaline, which in turn lead to a misorchestration of autonomic responses (Ainslie et al., 2007). To our knowledge, there is only one study that directly compared OI vs. OR during hypoxic tilt table test. That study found that OI is due to failure of vascular adjustment during hypoxic orthostatic stress, and not due to baroreflex misorchestration (Halliwill and Minson, 2005). Furthermore, 70% of that cohort remained OR, which is directly opposite of our findings, although both study protocols were radically different, so a direct comparison cannot be made. In this study, it appeared that the OI group expressed a dramatic increase, albeit unsuccessful, in SVRI during P2 which was not a successful strategy for maintaining MAP. It appears that CI is decisive in maintaining critical MAP during hypoxic orthostatic stress.

### Baseline Factors

At baseline, the OR group had a greater BMI, body weight, and Sp02p, with a lower HR as well as a prolonged RRI than the OI group. These findings strengthen the evidence that anthropometric factors such as BMI and body weight are associated with OR (Halliwill and Minson, 2005; Nordine et al., 2015; Christou and Kiortsis, 2017). While both BMI and body weight showed a high sensitivity and moderate PPV, the specificity was low. The physiological basis as to why an increased body size contributes to OR is due to greater and cardiac size and contractility function (Dewey et al., 2008).

The significant baseline HR difference between both groups could indicate discrepancies in hydration status, or baseline autonomic excitability. Prior investigations have also found that a higher resting HR is associated with OI (Lee et al., 2013). The high HR amongst the OI group may have been due to anxiety/excitability prior to the exposure. A heightened level of anxiety would have led to a pre-mature increase in sympathetic activation, thereby contributing to cardiac reserve exhaustion prior to P2. The presence of a longer RRI in the OR group would suggest decreased autonomic activity at baseline, which would support the previous statement. A previous study examining high/low tolerance to −60 mmHg LBNP found that high tolerant subjects had a significantly longer RRI vs. low tolerant participants, while high tolerant individuals had a lower baseline HR (Hinojosa-Laborde et al., 2011). Our study supports these prior results, showing that baseline HR and RRI offer valuable predictive information regarding baseline autonomic activity and OI/OR risk Also, although not tested prior to the study, dehydration or low plasma volume may have contributed to the baseline tachycardia, although no difference in SVI or CI were apparent. The significant difference in Sp02p between both groups may further indicate that the OI group had suboptimal hydration status, as a lower Sp02 may reflect this (Secher and Van Lieshout, 2005). Between these two factors, HR had a higher odd’s ratio than Sp02p, and thus may be more of a useful baseline predictor of OR/OI status.

### Orthostatically Resilient vs. Orthostatic Intolerance: Cardiovascular and Oxygenation Strategies During P1

During P1, the key cardiovascular differences between OR and OI were the change in CI, SVI, and SVRI. Specifically, the OR group responded to hypoxia via increasing CI, preserving SVI, increasing HR, while decreasing SVRI. This type of cardiovascular response during hypoxia would increase blood flow and ensure adequate cerebral oxygenation. The inability for the OI group to preserve SVI during this during hypoxia, would lead to a decrease in blood flow, and possible compromised tissue oxygenation. Also, as the OI group began with an elevated resting HR, the increases in HR during P1, may have further compromised SVI, thereby necessitating additional SVRI support. A decrease in SVI during hypoxia may also indicate reduced venous return (Siebenmann and Lundby, 2015), thereby triggering compensatory SVRI activity. The inability to decrease SVRI would further lead to additional 02 usage in the periphery thus further limiting available 02 reserves to the cerebral and myocardial tissues. Amongst these cardiovascular factors, the change in SVI during hypoxia exhibited the highest sensitivity, specificity, and PPV compared to delta CI and SVRI.

Cerebral oxygenation (TOIc) decreased and dHbc increased significantly amongst both groups, however, the OI group exhibited a significant decrease in 02Hbc. The reduction in 02Hbc and increase in dHbc amongst the OI group would suggest either an increase in cerebral 02 extraction, a reduction in cerebral 02 supply, or a combination of both. Given the significant decrease in SVI during P1, this may have led to a decrease in cerebral 02 supply, which may have triggered a greater increase in 02 extraction, thus increasing dHbc. The counter-measures enacted by the OI group were not sufficient enough to maintain cerebral oxygenation, while it appears that cerebral auto regulation was left largely unaffected. This trend of cerebral oxygenation in the OI group may have increased the risk for OI during subsequent orthostatic challenge. While no exact comparable study could be found to expand on this finding, prior results have demonstrated a link between sympathetic failure and falling cerebral oxygenation (Harms et al., 2000), which could indicate that the falling cerebral oxygenation in the OI group contributed to the cardiovascular collapse in P2. Clearly, further studies of this nature need to be performed to examine the link between OI and 02Hbc.

Prior evidence suggests that increases in cerebral blood flow during normobaric hypoxia may operate independently of respiratory or systemic cardiovascular reactions (AlSalahi et al., 2021). Other groups have not observed increased cerebral blood flow during hypoxia, suggesting that autoregulation remains intact (Cheng et al., 2017; Ogoh et al., 2018; van Helmond et al., 2018). Wilson et al. (2011) performed a multifaceted study examining cerebral oxygenation and blood flow during simulated and real-world hypoxic environments and found that at moderate hypoxia (Sp02 85% equivalent to 4250 meters of altitude), cerebral oxygenation significantly decreases, while at the same time, cerebral perfusion, and 02 delivery are not compromised. While doppler was not used in this study, no significant increase in tHbc could be found amongst our cohort, hinting that cerebral auto regulation remained largely intact during P1. The increase in dHbc amongst the OR group, while maintaining 02Hbc, would suggest an optimal cerebral 02 extraction and optimal 02 delivery, while a non-significant increase in tHbc would indicate a stable autoregulation during P1. In summary, cerebral oxygenation did not differ between groups, with only OI showing a significant 02Hbc decrease amongst the OI group, which may have contributed to cardiovascular collapse in P2.

In the peripheral tissues, changes in oxygenation were not significantly different between groups, however, group specific reactions were seen. TOIp decreased in both groups, however, this decrease was significant in the OI group, suggesting either a decrease in 02 flow to the periphery or increased 02 usage. Our evidence hints to an increased 02 utilization in the peripheral tissues amongst the OI group, as evidenced by a significant dHbp increase. An increase in dHbp would equate to increased 02 utilization. Local tissue hypoxia may have been further exacerbated by the SVI decrease amongst the OI group, which should have led to a refractory decrease in vascular activity (Hansen et al., 2000) however, this did not occur.

These findings can be summarized as the following: as the supply of 02 decreases during normobaric hypoxia, delivery of 02 to the critical organ systems is reliant upon increasing flow (Kane et al., 2020), via selective peripheral vasodilation, increasing CI via careful increases in HR, while maintaining SVI. In response to a falling 02, the OR group was able to down-tune SVRI, and increase CI (flow), thereby ensuring cerebral 02 supply, and minimizing excess 02 consumption in the peripheral tissues.

Ainslie et al., found that during acute normobaric hypoxia (Fi02 12%), 02Hb decreased, and dHb increased to a greater extent in the frontal cortex than in the vastus lateralis muscle, however, they found no difference in total hemoglobin change (Ainslie et al., 2007). These same trends were seen in our study, which would suggest that cerebral 02 supply and extraction are increased during hypoxia to shield the brain from hypoxic damage. The increase in tHbc amongst both groups suggest cerebral vasodilation, compared to the peripheral tissues, which runs in contrast to the findings of Ainsle.

Although no differences between groups with regards to central and peripheral oxygenation were observed, performance diagnostics for within group oxygenation patterns revealed that only dHbp did show a high sensitivity for OR/OI prediction, however, with a specificity of 0.0, and a PPV of 0.71. This suggest that rate of peripheral 02 utilization may have limited utility in revealing the propensity for an orthostatic event. A higher utilization of 02 during hypoxia could also be due to individual metabolic states, as well as a higher local metabolic rate in these regions, for example increases in vascular activity. Prior studies have suggested the use of peripheral oxygenation as an early detection system for OI predisposition during hypoxia (Soller et al., 2008; Rupp et al., 2013; Ovadia-Blechman et al., 2015; Schlotman et al., 2020). Based on the results of our study, the use of central and peripheral oxygenation via NIRS, especially dHb, may be used to pinpoint differences between high and low tolerant individuals during hypoxic orthostatic challenges, however, the predictive power is minimal.

### Orthostatically Resilient vs. Orthostatic Intolerance: Cardiovascular and Oxygenation Strategies During P2

During P2, the key differences between groups was that the OR group exhibited a greater MAP, SAP, DAP, and CI, and shortened RRI compared to the OI group, while no differences with regards to oxygenation were found. Despite significant decreases in SVI and CI during P2, increases in HR and SVRI were able to uphold critical MAP amongst the OR group, while these same increases in the OI group were not sufficient to maintain critical MAP. Although not specifically investigating hypoxic orthostatic stress, Convertino (2014), found that individuals exhibiting heightened increases in HR during orthostatic stress were more resilient, and that this increase in HR is associated with a greater sympathetic nervous system recruitment. The harnessing of sympathetic reserves could be supported by the significantly shortened RRI amongst OR, which would signal higher autonomic functioning. Furthermore, Hachiya et al. (2010), found that successful OR strategies involve delayed activation of SVRI, and gradual HR increases during graded orthostatic stress. While not directly comparable to our study, the OR group did show a heightened HR response during P2 and did exhibit a delayed SVRI activation. Therefore, we can confirm the findings from these studies, that successful cardiovascular OR strategies involve the harnessing of HR reserves, and delaying SVRI activity, either in normoxic or hypoxic orthostatic challenges. Davis et al. (2013), found that the ability to remain OR during hypoxic stand-test is dependent upon HR increases to maintain MAP. Work done by Fox et al. (2006), suggested that hypoxia exposure increases baroreflex sensitivity during LBNP, and that this advantage is expressed in individuals with innate hypoxic tolerance. Baroreflex sensitivity amongst the OR cohort may in fact have been heightened during P1, although this was not measured in this study. An increased baroreflex sensitivity as a response to acute onset hypoxia is known to occur, and is independent of respiratory factors (Halliwill et al., 2003). Furthermore, hypoxia can initiate full sympathetic activation, while withdrawing parasympathetic function (Halliwill and Minson, 2002; Blaber et al., 2003). The OR group may have exhibited a timely and balanced sympathetic activation during P2, whereas the OI group seems to have had a premature maximal sympathetic activation in P1, leading to a sympathetic overreaction, and collapse in P2. Furthermore, based on performance diagnostics, SAP showed the highest sensitivity, specificity, PPV, and odd’s ratio, compared to MAP, DAP, and CI. SAP is influenced by factors such as blood volume, SVI, and CI, and cardiac contractility. Although blood and plasma volume were not measured in this study, the increased CI in the OR group contributed to higher SAP in this study, as well as optimal cardiac contractility. Furthermore, SAP has been shown to be linked to higher BMI, and body weight, which may have further contributed to OR status (Das, 2017).

Cerebral oxygenation parameters, such as TOIc, and 02HBc further decreased significantly from baseline amongst both groups, while dHBc further increased. No differences in cerebral oxygenation could be pinpointed between both groups, meaning that cerebral oxygenation index during P2 did not play a role in OR/OI status in this study. The decisive factor for OR/OI is due to the maintenance of cerebral perfusion pressure, which is strongly correlated to maintaining MAP at a critical threshold (Kharraziha et al., 2019). Cerebral blood flow velocity is impaired in syncopal astronauts upon return to Earth under normoxic conditions, and although not measured in our study, the significant decrease in MAD, SAP, and CI, may have decreased cerebral blood flow velocity to a critical degree amongst the OI group (Blaber et al., 2011). Although not directly comparable to this study, similar patterns of cerebral oxygenation were found during G-Force exposure via human centrifuge under normoxic conditions, and that these decreases in cerebral oxygenation did not correlate with cortical activity (Smith et al., 2013). A loss of cerebral perfusion pressure may also be due to complete sympathetic failure (Harms et al., 2000). Cognitive ability during hypoxic hyper-gravity may not be solely dependent upon cerebral oxygenation, the maintenance of cerebral perfusion pressure would be paramount for crew health.

The significant increase in tHbp, 02Hbp, dHbp, and decrease in TOIp were not different between groups, and are a result of significant venous pooling during LBNP. At a level of −70 mmHg, a cranial to caudal shift of 25% of blood volume is expected (Hinojosa-Laborde et al., 2014). Also, despite a significant degree of venous pooling, arteriolar vasoconstriction is not impaired at this level of orthostatic stress (Habazettl et al., 2015).

It appears from our data, that a successful OR strategy during combined hypoxic orthostatic stress relies on mobilizing sympathetic reserves (RRI, HR, and SVRI), during a significant fall in blood volume, which maintains CI, thus contributing to the maintenance of stable MAP and SAP. Secondarily, it appears that a delayed and balanced SVRI, combined with this HR response, can maintain MAD, SAP, and DAP at critical levels which maintains cerebral perfusion pressure. An exaggerated SVRI response, amongst the OI group was not sufficient to maintain MAP, despite further increases in HR. Based on these results, OR is dependent upon the maintenance of cerebral perfusion, rather than that of cerebral oxygenation (Kharraziha et al., 2019). Finally, it appears that the change in SAP during P2 was strongly associated with LBNP OR status, and that SAP may contribute to maintaining cerebral perfusion more than MAD.

### Limitations

Despite having a good sample size and being only one of a few studies that directly examined the differences between OR and OI subjects, there are a few limitations to this study. Primarily is the omission of respiratory parameters. Respiratory rate, tidal volume, and minute ventilation were unfortunately not measured. An increase in minute ventilation is expected during hypoxia, however, this change in ventilation does not seem to influence cerebral perfusion (AlSalahi et al., 2021). Also, no comparison was performed using normoxic LBNP. A cross-over design could have directly compared LBNP responses to the 2 conditions (normoxic and hypoxic). One study found that the HR response during a normoxic vs. a hypoxic orthostatic challenge did not differ (Koelwyn et al., 2013), and that during any orthostatic challenge, the baroreceptor response overrides the peripheral chemoreceptor response (Shepherd et al., 2020). To solidify this finding, a cross-over study design would have been useful. Another drawback is the homogenous pool of exclusively men. Aerospace crews are composed of women and men, and gender specific countermeasures will be needed, as there appears to be a varying gender specific response to orthostatic stress (Barnes and Charkoudian, 2020). Another limitation is the use of NIRS for measuring cerebral and peripheral oxygenation. NIRS, although a useful instrument, can relay information only regarding the change in oxygenation, and not perfusion. To better solidify the findings of our study, doppler images of the middle cerebral artery could have been obtained to estimate the degree of cerebral perfusion. Also, it is difficult to estimate if our NIRS recordings reflected a more venous or arterial aspect of oxygenation. Recent evidence suggests that NIRS is composed of 50% arterial, and 50% venous concentrations, as opposed to the previously suggested 25% arterial, and 75% venous (Sørensen et al., 2014). And finally, no serum values of hemoglobin, noradrenaline, adrenaline, creatinine, of hematocrit were analyzed. Prior reports have suggested that OF is associated with excess serum adrenaline during simulated hypoxic hypovolemia (Crandall et al., 2019). Given the scant number of studies on this topic, we missed an opportunity to verify this evidence. Hemoglobin can play a role in the hypoxic response, and although we did not measure hemoglobin, one can assume that any symptoms of anemia would have been detected during the rigorous military pilot program. Hematocrit and creatinine were not measured at baseline, and thus, there is speculation as to the hydration status of our cohort. Dehydration can affect the response to hypoxia (Richardson et al., 2009), as well as tolerance to an orthostatic stimulus (Schroeder et al., 2002). Surrogate baseline factors such as an elevated HR and decreased Sp02d may suggest that the OI group began the run in a dehydrated state, however, this is purely speculation.

## Conclusion

In conclusion, successful OR strategies during SHHOC depend upon baseline anthropometric (greater body weight and BMI), lower baseline HR with higher RRI and Sp02p. Furthermore, responding to hypoxia via elevations in CI, maintaining SVI, and decreasing SVRI, with the minimization of central/peripheral 02 usage is also a key strategy. During hypoxic orthostatic challenge, a mobilization of HR reserves and delayed SVRI activation, leading to the maintenance a higher CI which maintains MAP, DAP, and SAP above critical levels, are key OR strategies. The utilization of central and peripheral oxygenation parameters did not reveal any differences between OR/OI during the SHHOC, although inter-group trends did show a trend amongst the OI group for higher peripheral 02 utilization/extraction during hypoxia along with a decrease in cerebral 02 supply.

The results of this study can be useful for developing effective non-invasive monitoring strategies for future aerospace crews and for establishing crew medical support to pinpoint which crew members are predisposed to an orthostatic event during critical periods such as acute atmospheric changes combined with increases in gravitational vector. Maintaining body weight, and the use of SVI tracking may enhance crew-health to prevent an orthostatic event, and better ensure the continued success of human aerospace travel.

## Data Availability Statement

The original contributions presented in the study are included in the article/supplementary material, further inquiries can be directed to the corresponding author/s.

## Ethics Statement

Ethical review and approval was not required for the study on human participants in accordance with the local legislation and institutional requirements. The patients/participants provided their written informed consent to participate in this study.

## Author Contributions

MN, HH, H-CG, and OO devised, financed, and performed the study. PD and JP supervised and assisted in performing the study. KB performed statistical analysis. MN, OO, and ST wrote the manuscript. All authors contributed to the article and approved the submitted version.

## Conflict of Interest

The authors declare that the research was conducted in the absence of any commercial or financial relationships that could be construed as a potential conflict of interest.

## Publisher’s Note

All claims expressed in this article are solely those of the authors and do not necessarily represent those of their affiliated organizations, or those of the publisher, the editors and the reviewers. Any product that may be evaluated in this article, or claim that may be made by its manufacturer, is not guaranteed or endorsed by the publisher.
